# The fertilization-induced zinc spark is a novel biomarker of mouse embryo quality and early development

**DOI:** 10.1038/srep22772

**Published:** 2016-03-18

**Authors:** Nan Zhang, Francesca E. Duncan, Emily L. Que, Thomas V. O’Halloran, Teresa K. Woodruff

**Affiliations:** 1 Department of Obstetrics and Gynecology, Feinberg School of Medicine, Northwestern University, Chicago, IL 60611, USA; 2 The Chemistry of Life Processes Institute, Northwestern University, Evanston, IL 60208, USA; 3 Department of Chemistry and Department of Molecular Biosciences, Northwestern University, Evanston, IL 60208, USA

## Abstract

Upon activation, mammalian eggs release billions of zinc ions in an exocytotic event termed the “zinc spark.” The zinc spark is dependent on and occurs coordinately with intracellular calcium transients, which are tightly associated with embryonic development. Thus, we hypothesized that the zinc spark represents an early extracellular physicochemical marker of the developmental potential of the zygote. To test this hypothesis, we monitored zinc exocytosis in individual mouse eggs following parthenogenetic activation or *in vitro* fertilization (IVF) and tracked their development. Retrospective analysis of zinc spark profiles revealed that parthenotes and zygotes that developed into blastocysts released more zinc than those that failed to develop. Prospective selection of embryos based on their zinc spark profile significantly improved developmental outcomes and more than doubled the percentage of embryos that reached the blastocyst stage. Moreover, the zinc spark profile was also associated with embryo quality as the total cell number in the resulting morulae and blastocysts positively correlated with the zinc spark amplitude (R = 0.9209). Zinc sparks can thus serve as an early biomarker of zygote quality in mouse model.

Recently, zinc emerged as an essential element required for the completion of meiosis and egg activation^1^,^2^,^3^,^4^,^5^,^6^,^7^,^8^,^9^,^10^,^11^. In mouse, meiotic maturation is accompanied by a substantial (50%) increase in total zinc content^4^, which is required to transition through anaphase. At fertilization, total zinc levels decrease. Within minutes of fertilization, zinc is released from the zygote into the extracellular space in a secretory event termed the “zinc spark”^3^,^7^. This zinc release closely follows calcium transients and is necessary for cell cycle resumption via pathways that include modulation of the cell cycle regulatory protein EMI2^2^,^8^. Our work has shown that in human (Duncan *et al*. a manuscript submitted to *Scientific Reports*) and other mammalian species^12^, zinc spark is a hallmark of egg activation that happens upon parthenogenetic activation or fertilization^13^. Thus, the zinc spark represents an early marker of the quality of the mammalian egg, one of particular interest because it can be measured extracellularly. The predictive quality of the zinc spark as a marker of the developmental potential of human zygotes, however, is not readily evaluated due to ethical, legal and technical considerations regarding human embryo research. We therefore used a mouse model to determine whether zinc spark profiles are correlated with preimplantation embryo development^14^,^15^,^16^. We measured zinc spark profiles in individual mouse eggs following parthenogenetic activation or *in vitro* fertilization (IVF) and tracked the development of the resulting parthenotes and embryos, respectively. We found a significant correlation between the amplitude of the fertilization-induced zinc spark and successful embryo development to the blastocyst stage. Prospective selection of zygotes based on their zinc spark amplitude improved IVF outcomes and more than doubled the blastocyst percentage, indicating that the magnitude of zinc released at the time of fertilization can be used to predict embryo quality. These studies provide strong evidence that the zinc spark, a conserved hallmark of egg activation in multiple mammalian species including human, can be used to predict IVF outcomes.

## Results

### Distinct ionomycin-induced zinc spark profiles are correlated with egg activation and blastocyst formation

**Figure 1:**
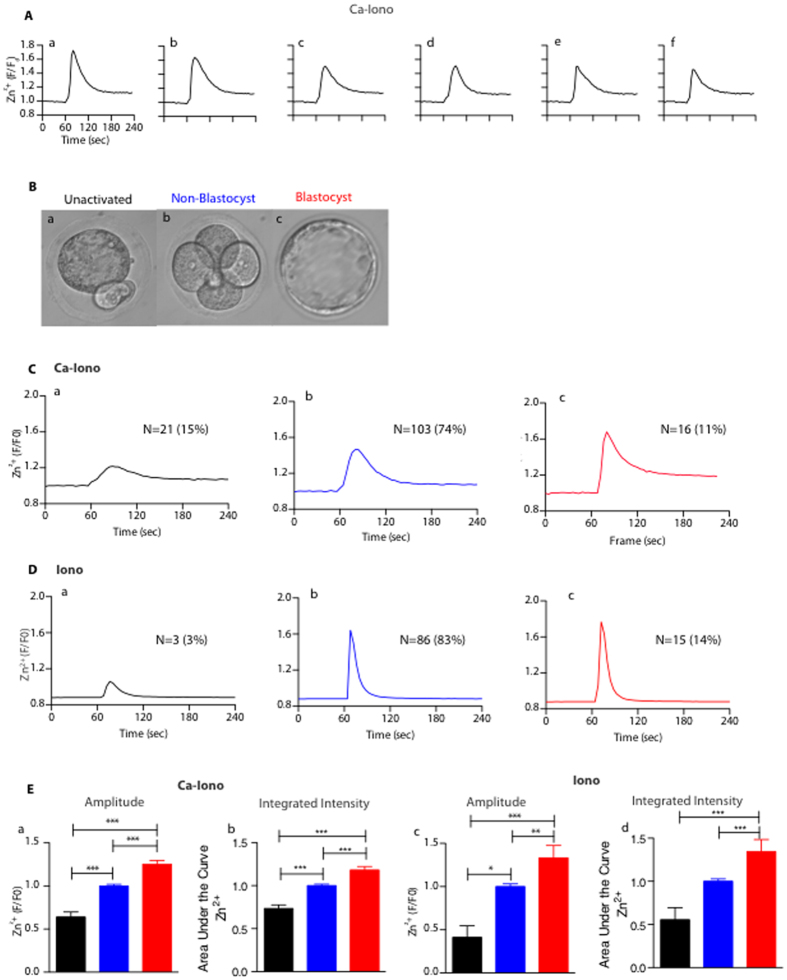
Distinct Ionomycin-induced zinc spark profiles are correlated with egg activation and blastocyst formation. (**A**) Representative time traces of normalized zinc fluorescence (F/F_0_) following activation by 5 μM Ca-Iono. F_0_ was calculated by averaging the first five fluorescence measurements for each egg prior to zinc spark. All six eggs (a-f) were collected from the same individual mouse. (**B**) Representative images of unactivated eggs (a, black color), non-blastocysts (b, blue color) and blastocysts (c, red color). (**C**) Representative profiles of Ca-Iono induced zinc sparks in the eggs that showed differential developmental outcomes: (a) unactivated, (b) non-blastocyst, and (c) blastocyst. (**D**) Representative profiles of Iono induced zinc sparks in the eggs that showed differential developmental outcomes: (a) unactivated, (b) non-blastocyst, and (c) blastocyst. (**E**) Amplitude (a,c) and integrated intensity (b,d) of the Ca-Iono (a,b) or Iono (c,d) induced zinc sparks in unactivated eggs (black), non-blastocysts (blue) and blastocysts (red). The non-blastocyst group was normalized to one and values for the other groups are presented relative to that. (*p < 0.05; ** p < 0.01; ***p < 0.001).

To determine whether there is variability in zinc spark profiles that could underlie differences in the quality of individual eggs, we imaged zinc sparks in eggs collected from the same animal following activation with 5 μM Ca-ionomycin (Ca-Iono). Ca-Iono directly delivers exogenous calcium into the cell and induces a single large intracellular calcium transient^17^. Consistent with our previous findings that a rise in intracellular calcium triggers the zinc spark^12^, each of the cells treated with Ca-Iono mounted a single zinc spark (Fig. 1A a-f and Movie S1). Of note, each egg had a distinct zinc spark profile, which can be analyzed using a number of parameters including amplitude (maximum peak height), integrated intensity (area under the curve), duration, and rate of rise calculated as amplitude divided by the rising time (Fig. S1).

To further test the relationship between zinc spark profile and mouse embryonic development, we analyzed zinc spark profiles in individual eggs following parthenogenetic activation in calcium-free media using Ca-Iono or the apo (i.e. calcium free) form of ionomycin (Iono)^18^. Both reagents can induce an intracellular calcium rise. However, Ca-Iono delivers a bolus of exogenous calcium directly into the egg therefore Iono is thought to provide a better readout of egg quality as it only triggers the release of endogenous calcium stores to mount the activation-associated calcium transients. Cells treated with either activating agent were then allowed to develop, and at the conclusion of the experiment were characterized as “unactivated” eggs (cells that remained at metaphase of meiosis II; MII), “non-blastocyst” parthenotes, and “blastocyst” parthenotes (Fig. 1B). We found that compared to Ca-Iono, Iono treatment yielded higher percentages of both egg activation (85% Ca-Iono, 97% Iono; Fig. 1C,D) and blastocyst formation 120 hours post activation (11% Ca-Iono, 14% Iono; Fig. 1C-c,D-c). This incidence of blastocyst formation is comparable to the rate reported in the literature (16%) using the same activation method^19^. A majority of the eggs (74% Ca-Iono, 83% Iono; Fig. 1C-b,D-b) initiated preimplantation embryo development but did not reach the blastocyst stage (“non-blastocyst”).

Using this data, we performed a retrospective analysis to characterize the zinc spark profile associated with each of the three developmental outcomes. In both Ca-Iono and Iono treatments, the unactivated eggs displayed significantly lower zinc spark amplitudes and integrated intensity of all the groups (Fig. 1E). Zinc sparks in eggs that developed into blastocysts had higher amplitude and larger integrated intensity compared to non-blastocysts (Fig. 1E). We note that the duration of the Ca-Iono induced zinc spark was longer than that induced by Iono, which might be an effect of the excess calcium introduced into the cell by the Ca-Iono. Taken together, these results indicate that zinc spark profiles are closely related to egg quality and parthenogenetic development in mice.

To further test these findings, we prospectively selected embryos based on their zinc spark profile and monitored their development. Because the correlation between zinc spark profiles and embryo quality is more robust in Ca-Iono method compared to Iono method, we activated eggs with Ca-Iono and separated and cultured the parthenotes in two groups based on their zinc spark amplitude: those in the top 50^th^ percentile and those in the bottom 50^th^ percentile. These were then scored on their ability to progress to the blastocyst stage. Published rates of blastocyst formation for this protocol are low (16%)^19^ but we find more than twice as many activated eggs in the group whose zinc spark amplitude was in the top 50^th^ percentile progressed through early development (top 50^th^: 19% blastocyst, bottom 50^th^: 8% blastocyst). These results indicate that the zinc spark profile is a statistically significant marker of mammalian embryo quality that can be used to select embryos with greater developmental potential.

### Zinc sparks induced by fertilization correlate with development to blastocyst stage

**Figure 2:**
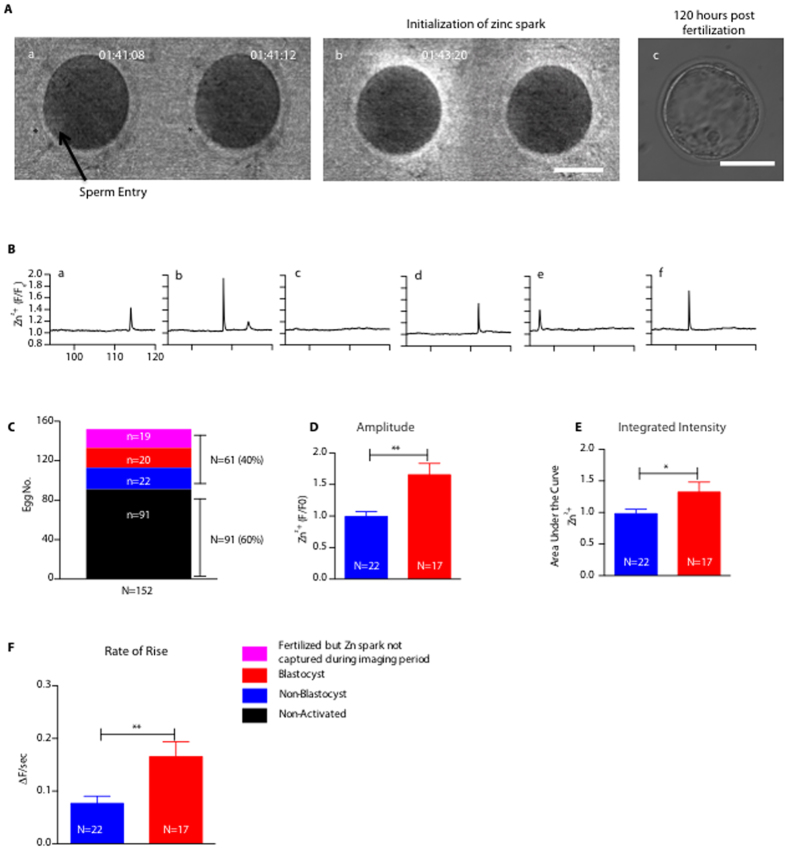
Larger zinc spark release during IVF is associated with embryos that develop to the blastocyst stage. (**A**) (a) Representative images of a MII egg being binding a single sperm. Asterisks and an arrow denote the location where the sperm entered the egg; (b) the egg released zinc shortly after the sperm entry; (c) image of the same fertilized eggs 120 hours post zinc imaging. (**B**) Representative time traces of normalized zinc sparks (F/F_0_) in mouse eggs upon fertilization. (**C**) Statistical summary of the outcomes of the imaged eggs. (**D**) Comparison of amplitude of zinc sparks in the non-blastocyst and blastocyst embryos. (**E**) Comparison of the integrated intensity of zinc sparks in the non-blastocyst and blastocyst embryos; (**F**) Comparison of the rate of rise of zinc sparks in non-blastocyst and blastocyst embryos. (*p < 0.05; **p < 0.01; ***p < 0.001).

We next investigated if zinc spark profiles observed during IVF were correlated with embryo development. We imaged a group of zona pellucida (ZP) intact eggs during fertilization and tracked their long-term development (N = 152, Fig. 2C). Among these eggs, we were able to document the zinc spark in real time for the first time upon fertilization. This egg mounted a zinc spark exactly two minutes and eight seconds after the sperm penetrated the ZP, and it reached the blastocyst stage 120 hours after fertilization (Fig. 2A and Movie S2). As was observed with parthenogenetic activation, IVF elicited different zinc spark profiles among eggs collected from the same animal. Figure 2B shows six fertilized eggs – four had one zinc spark (a,d,e,f); one had two zinc sparks (b) and one egg did not release a zinc spark (c) during the two-hour monitoring window.

Under the experimental IVF imaging conditions, 61 out of 152 eggs (40%) were successfully fertilized as confirmed by pronucleus (PN) formation and further embryo development. A portion of these eggs (42 out of 61) was fertilized within the two-hour imaging window and zinc sparks were observed (Fig. 2C). However in the remaining fertilized eggs, zinc sparks were not observed, likely due to the cells being fertilized after the imaging window as zinc imaging started immediately after the addition of sperm (Fig. 2C). The remaining eggs did not produce zinc sparks and showed no signs of egg activation. Of the 42 eggs that displayed zinc sparks and showed signs of egg activation, 35 eggs released a single zinc spark and 7 eggs exhibited two to four zinc sparks. To determine the relationship between the fertilization-induced zinc sparks and developmental potential, we analyzed the profile of the first zinc spark in all embryos. The embryos that developed to the blastocyst stage had a higher zinc spark amplitude (P < 0.01, Fig. 2D) and larger integrated intensity of zinc release compared to non-blastocyst embryos (P < 0.05, Fig. 2E). We also found that the rate of rise of the zinc spark was significantly higher in blastocyst embryos (P < 0.01, Fig. 2F). Altogether, these results further indicate that zinc spark profiles can be correlated with embryonic developmental potential in the context of fertilization.

### Zinc spark amplitude positively correlates with total cell number of late stage pre-implantation embryos

**Figure 3:**
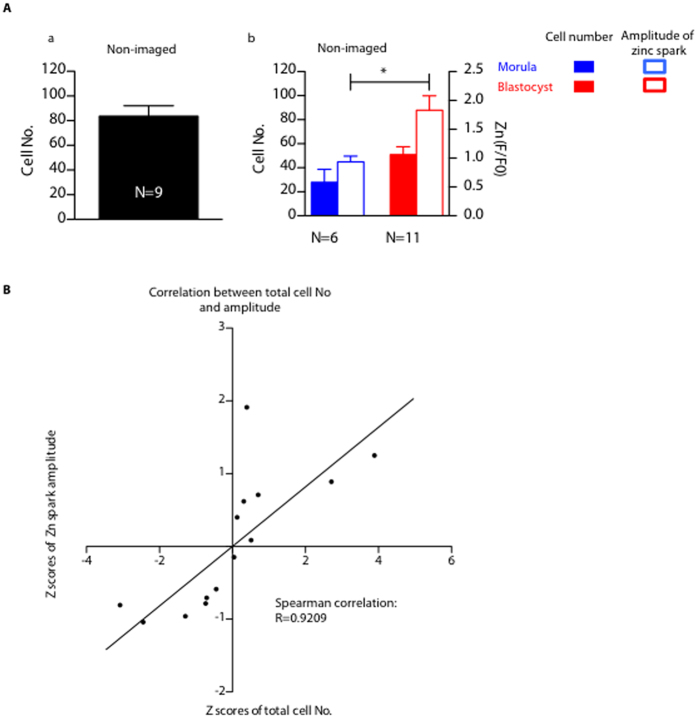
Zinc sparks are highly correlated with the total cell number of late stage pre-implantation embryos. (**A**) (a) Cell numbers of the embryos that were not imaged during fertilization (non-imaged); (b) amplitude of zinc sparks (empty bars) and total cell numbers (solid bars) in the embryos that arrested at morula stage (blue) or reached blastocyst stage (red) 120 hours post zinc imaging. (**B**) Z scores, which is calculated as (x-xbar)/SD, of total cell number of embryos and the z scores of the zinc spark amplitude in the imaged embryos from (**A**) are highly correlated. (*p < 0.05, ***p < 0.001).

To investigate whether the zinc spark profile is associated with embryo quality in addition to developmental progression, we next compared the fertilization-induced zinc spark profile to the total cell number of embryos that progressed to the morula or blastocyst stages because this parameter is tightly correlated with embryo quality^20^. We found that morulae and blastocysts had ~30 and 50 cells, respectively, following zinc spark imaging and culture for 120 hours (Fig. 3A-b). This is in contrast to >80 cells in control embryos that had not been imaged during activation (Fig. 3A-a). Although these findings indicate that the imaging itself impacts embryo development, embryos that reached the blastocyst stage had significantly higher amplitude zinc sparks compared to the embryos that arrested at the morula stage (P < 0.05, Fig. 3A-b). Importantly, for each individual embryo, we plotted the Z score (a measure of standard deviation)^21^ for zinc spark amplitude vs. the Z score for total cell number and fit this data using linear regression analysis (Fig. 3B). We found that the Spearman correlation coefficient between these two variables is close to 1 (R = 0.9209, Fig. 3B) indicating that the zinc spark amplitude induced by fertilization is highly correlated with the cell number of embryos several days later.

## Discussion

Today the use of Assisted Reproductive Technologies (ART) plays a prominent role in fertility treatment^22^. To increase the rate of successful ART cycles, clinical practice relies on extended embryo culture and multiple embryo transfers. However, both methods have their limitations^23^. Long-term culture can have quantifiable impacts on gene expression, imprinting, and behavior in the resulting offspring^24^,^25^,^26^,^27^. Multiple embryo transfer tends to cause multiple gestations, leading to a myriad of maternal (i.e. miscarriage, pre-eclampsia, gestational diabetes, pre-term birth) and fetal (i.e. developmental defects) complications^23^,^28^. Therefore, there is an urgent and unmet need to move the field towards the goal of single embryo transfer. Developing non-invasive methods to select the highest quality embryos at a very early stage of development is a promising solution^29^. This study examined the relationship between the activation-induced zinc spark profile and embryo development in mouse, an analysis that cannot be conducted with human eggs in a research setting using United States federal funds^14^,^15^,^16^. Using parthenogenetic activation and IVF we found that parthenotes and embryos with better developmental outcomes (i.e., development to blastocyst stage) released zinc sparks with higher amplitude and integrated intensity compared to embryos of lower quality (unactivated eggs and those that did not reach the blastocyst stage). Moreover, the zinc spark amplitude significantly correlated with the cell number of late-stage pre-implantation embryos (morulae and blastocysts). Collectively, these findings suggest that the zinc spark is not only a hallmark of egg activation in human (Duncan *et al*. accompanying manuscript) and mouse but that the magnitude of zinc release as detected by these fluorescence microscopy methods is closely correlated with embryo quality. These results indicate that preimplantation embryo developmental outcomes can be predicted by selecting embryos based on the zinc spark amplitude or potentially by other quantitative measurement of zinc release. It is worth noting that the eggs were exposed to laser scanning and zinc dye-containing medium for 2 hours during zinc imaging. The adverse effects of the operation led to the production of embryos that contained fewer cells at the blastocyst stage than the controls that had not been imaged. Despite this current limitation, embryos with high and low zinc amplitude and integrated intensity could be directly compared to each other as case and control groups. Within these two groups, the data show that developmental outcomes are positively correlated to zinc physiology. Future studies will require the invention of a non-invasive zinc imaging method to further test the utility of zinc release to developmental outcomes including live birth. Ideally, a non-invasive imaging method would avoid introducing any hazardous factors that disturb the physiological condition of the fertilization medium. These studies that will minimize dye and laser exposure to eggs or embryos are underway.

Capturing the moment when a sperm enters an egg in real time is technically challenging as the sperm can approach the egg from any direction at any time and the mammalian egg is approximately 100,000 times the volume of a single sperm cell^30^. Therefore it is very rare for a single sperm entry site to coincide with the focal plane of the microscope. Here we captured the moment of fertilization, which corresponded with zinc release at 2 min and 8 sec after the sperm binding to the ZP. Although the sample size is unique (i.e., N = 1), this observation is consistent with the timing of the first intracellular calcium transient, which initiates at the site of sperm entry ~12 sec after the sperm interacts with the ooplasmic membrane^31^. The zinc spark occurred as a bright fluorescent signal at the hemisphere opposite the sperm entry site. This is consistent with the distribution of zinc vesicles in the cortical area and further supports the notion that these “zinc loaded” vesicles located in the cell periphery are the source of the zinc spark^7^. However, we could not determine the precise sperm entry site (i.e. microvillar vs. amicrovillar domain) due to the two-dimensional nature of the image.

In IVF experiments a majority of fertilized cells exhibited one zinc spark during the 2-hour imaging period. This is echoed by our recent finding that PLCzeta cRNA injection, which is the physiological signal that best mimics fertilization, induced a maximum of one zinc spark in human eggs (Duncan *et al*. a manuscript submitted to Scientific Reports). Although we cannot exclude the possibility that subsequent zinc sparks occurred beyond the 2-hour imaging window, this is unlikely because the amplitude of the intracellular calcium transients, a well-known zinc spark trigger, gradually decline to zero after their initiation (Fig. S2B)^32^. We also observed multiple zinc sparks during fertilization in a small fraction of the eggs (7 of 42). This data suggests that there exists heterogeneity in the number of zinc sparks induced by physiological stimulation. Interestingly we noted that the multiple zinc spark embryos had a higher blastocyst rate compared to the single zinc spark embryos (71% vs 41%). The precise relationship between the zinc spark number and embryo development are intriguing and warrant further investigation.

The findings that eggs that are able to release more zinc during activation are more likely to support a healthy and competent developing embryo can be interpreted from many different perspectives. One explanation is that the zinc spark is the major mechanism by which eggs export large amount of zinc during activation^4^,^7^,^12^, and the loss of intracellular zinc is necessary to mediate the egg-embryo transition through pathways that regulate the activity of Early Meiosis Inhibitor 2 (EMI2, official symbol FBXO43), which mediates cell cycle arrest^2^,^33^. Consistent with this notion, we found that when the effects of zinc exocytosis is counteracted by treating eggs with zinc pyrithione (ZnPT), a zinc ionophore that elevates the intracellular free zinc content of mammalian cells, egg activation was inhibited^12^. We have also found that zinc exocytosis contributes to zona hardening and the block to polyspermy (Que *et al*. submitted). Thus a number of molecular pathways and attributes of embryo physiology may be subject to modulation by zinc fluxes. A compromised zinc spark could be an indirect readout of poor cytoplasmic maturation in eggs. This notion is supported by our findings in human that there is a meiotic maturation-dependent acquisition of the ability of the egg to mount a zinc spark, with gametes arrested at prophase I having on average a smaller zinc spark compared to those at the metaphase II stage (Duncan *et al*. accompanying manuscript).

A holy grail in the field of Assisted Reproduction Technology is to identify reliable non-invasive and quantitative markers that can be used to predict embryo development; however, the selection of embryos is still mainly based on morphological observations, such as division rate, degree of fragmentation and blastocyst formation^34^. Recently, great progress has been made to identify a reliable marker of embryo quality. For example, the kinetics of early embryo development is closely related to blastocyst formation^35^, and the aneuploidy status of an embryo can be predicted by a transcriptomic analysis^36^. But these methods have their own limitations. For example, these methods require the embryos be cultured *in vitro* at least for 2 days. Embryo culture not only has negative impacts on embryo development but also has long-term developmental and behavioral consequences^37^,^38^. Our study, points toward a window of opportunity within minutes of fertilization that provide important insight into the developmental potential of the zygote. This could eliminate the need to culture embryos for an extended period of time. Moreover, improving methods for embryo selection could have implications not only for pregnancy outcomes but for embryo cryopreservation as well. The disposition of cryopreserved embryos is a major concern in the IVF field. Storing fewer embryos based on their zinc signature could reduce cost and angst associated with this issue.

Taken together, these two studies show that zinc is necessary to the transition of human eggs to parthenotes and points the way to further interrogation of the inorganic signals that underlie the meiosis-to-mitosis transition. Further, the studies suggest that a zinc profile could be used to accept or reject zygotes for further *in vitro* development or for cryopreservation^39^. To realize this potential, new technologies aimed at the measurement of zinc in the immediate post-fertilization media are under development. The new method will also be vigorously tested in more clinically relevant scenarios including ICSI.

## Materials and Methods

### Study design

A retrospective study was designed to evaluate the correlation between zinc spark profiles and embryo development in mouse. We performed an *in vitro* experiment that used gametes collected from 4–6 weeks old CD-1 mice. We performed a post hoc analysis and the sample size for mice (minimum n = 3) were based on those required to achieve the chosen statistical significance criterion. All post hoc quantitative analysis of zinc sparks was performed identically using Image J and Prism. No outliers were excluded.

### Animal care and welfare

Animals used for gamete collections herein were handled following the National Research Council’s Animal Care and Welfare Guidelines. These procedures were approved by the Institutional Animal Care and Use Committee (IACUC) at Northwestern University. All the experiments were carried out in accordance with the approved guidelines.

### Egg collection and culture conditions

To collect MII-arrested eggs, CD-1 female mice, 6–8 weeks old, were injected with 5 IU of pregnant mare serum gonadotropin (PMSG; Sigma, St. Louis, MO; all chemicals were purchased from Sigma unless otherwise specified). MII eggs were obtained from the oviducts 12–14 h after injection of 5 IU hCG, which was administered 46–48 h after PMSG injection. Eggs were released into HEPES-buffered tyrode-lactate solution (TL-HEPES) supplemented with 5% heat-treated fetal calf serum (FCS; Gibco BRL, Grand Island, NY) followed by treatment with 0.1% bovine testes hyaluronidase for 3–5 min to remove cumulus cells. MII eggs were thoroughly washed and transferred into 50 ml drops of KSOM (Potassium Simplex Optimized Medium; Specialty Media, Phillipsburg, NJ) containing 0.1% BSA under paraffin oil at 36.5 °C and in a humidified atmosphere containing 5% CO_2_ before imaging or activation treatment.

### Live cell fluorescence microscopy and MII eggs activation

For Ionomycin (Iono) activation experiment, mouse eggs were placed in a 360 μl drop of 20 μM FZ-3 (for zinc monitoring) in Ca-free hCZB medium under oil in a coverslip-bottom imaging dish (MatTek Corp, Ashland, MA). Imaging was performed at 37 °C on a TCS SP5 (Leica) confocal microscope (Leica Microsystems, Heidelberg, Germany) equipped with a stage top incubator (ToKaiHit, Shizuoka, Japan), 20× objective and an Ar (488 nm) laser line. Initial fluorescence images were obtained prior to activation. A 40 μl 10× stock solution of 50 μM Iono was introduced to the imaging drop 1 minute after the start of zinc imaging. Images were collected every 4 seconds for 5 minutes. Eggs were then kept individually in cycloheximide (10 mg/ml) and cytochalasin B (5 mg/ml) in KSOM (0.1% PVA) at 36.5 °C and in a humidified atmosphere containing 5% CO_2_ for 4 hr. After washing, each egg was transferred to individual KSOM droplets (20 μl) and evaluated using phase contrast microscopy for signs of activation and embryo development. The eggs that did not show pronuclear formation or underwent apoptosis within 8 hr post activation were defined as “unactivated eggs”. The activated eggs were divided into two groups according to their developmental outcomes at 120 hr: (1) the “non-blastocyst” group, consisted of embryos that were activated but not able to reach blastocyst stage; (2) the “blastocyst” group, consisted of embryos that were able to reach blastocyst stage 120 hr post activation.

In the Ca-Ionomycin (Ca-Iono) activation experiments, mouse eggs were placed in a 360 μl drop of 50 μM FlZ-3 in Ca-free hCZB medium under oil in a coverslip-bottom imaging dish (MatTek Corp, Ashland, MA). Imaging was performed at 37 °C on a EVOS cell imaging system (Life Technologies, Grand Island, NY) using a GFP light cube with 488 nm excitation or on a Nikon Eclipse system with 488 nm excitation. A 40 μl 10 x stock solution of 50 μM Ca-Iono was introduced to the imaging drop 1 minute after the start of zinc imaging. Images were collected every 4 seconds for 5 minutes. The eggs were then activated and cultured as described in the Ionomycin activation experiment.

For the IVF experiments, sperm from the cauda epididymides of proven breeder CD-1 males were collected and purified using a conventional swim up method^40^. Sperm capacitation happened in 2 hours in human tubal fluid (HTF) medium supplemented with 3 mg/ml bovine serum albumin (BSA, MP biomedicals, Solon, OH). Capacitated sperm were then added to the eggs to a final concentration of 1 × 10^5^ sperm/ml in the HTF medium containing 3 mg/ml BSA. The standard IVF process continued for 6 hours.

To image zinc sparks during fertilization, mouse MII eggs were allowed to settle in a 50 μl HTF drop that did not contain any serum on a glass-bottom dish (MatTek Corp, Ashland, MA). After 5 minutes, regular HTF medium that contained 3 mg/ml BSA and 23 μM FluoZin-3 (Invitrogen, Grand Island, NY) was slowly added to a final volume of 400 μl, taking care not to disturb the eggs. Capacitated sperm were added to the eggs to a final concentration of 1 × 10^5^ sperm/ml and image acquisition began immediately on a TCS SP5 confocal microscope using 488 nm excitation and an open pinhole. Images were collected every 4 seconds. We shortened the fertilization and imaging time from 6 hours to 2–3 hours to mitigate the damage caused by laser exposure and presence of zinc dye in the medium to both the egg and sperm. Eggs were then kept individually in droplets of potassium simplex optimized medium (KSOM, Millipore, Billerica, MA) and embryo development was evaluated at 8 hours and 120 hours post fertilization. As a control experiment to confirm the fertilizability of the eggs under the imaging condition, we also employed zona pellucida (ZP) free eggs for the zinc imaging and IVF. ZP was removed by brief treatment in acidic Tyrode’s solution (PH2.5, Millipore, Billerica, MA).

To image intracellular calcium and extracellular zinc simultaneously, ZP free eggs were first incubated with 1 μM Fluo4-AM (for calcium monitoring, Molecular Probes, Eugene, OR) and 0.02% Pluronic F-127 (Molecular Probes, Eugene, OR) in TL-HEPES for 20 minutes at 37 °C. Gametes were then washed through dye-free medium and placed in 20 μM FluoZin-3 in HTF medium as described above. Image analysis was performed by defining regions of interest (ROIs) and measuring fluorescence intensity over time using ImageJ^41^. Intracellular ROIs were drawn as the entire interior area of the cell. Extracellular ROIs were defined as a ring around the perimeter of the cell. The ring thickness was conserved for all data analyses.

### Immunofluorescence

Embryos were fixed in 3.8% paraformaldehyde (Electron Microscopy Sciences, Hatfield, PA, USA) in PBS for 1 hour at RT. The cells were then permeabilized in PBS containing 0.1% Triton X-100 and 0.3% bovine serum albumin (BSA) for 15 min at room temperature and rinsed twice through blocking solution of PBS containing 0.3% BSA and 0.01% Tween-20 (Sigma-Aldrich, St. Louis, MO). The eggs were mounted in Vectashield Mounting Media with DAPI (Vector Laboratories, Burlingame, CA, USA). Slides were examined on an SP5 confocal microscope with x40 oil-immersion objective, and near-UV (405 nm) laser lines. Z-stack images were obtained every 2 to 5 μm to span the entire volume of the embryo. Image analysis was performed using ImageJ to count the total cell number of each embryo.

### Statistical analysis

Values from three or more experiments, performed on different batches of eggs, were used for evaluation of statistical significance. The Prism5.0 software (Graphpad Software, La Jolla, CA) was used to draw graphs and perform the statistical comparisons using appropriate Student’s t-test, one-way ANOVA or Chi square analysis. Values are shown as means ± S.E.M, and significant differences were considered at *p* values < 0.05.

## Additional Information

**How to cite this article**: Zhang, N. *et al*. The fertilization-induced zinc spark is a novel biomarker of mouse embryo quality and early development. *Sci. Rep.* **6**, 22772; doi: 10.1038/srep22772 (2016).

## Supplementary Material

Supplementary Information
