# Supplementary material for: The fertilization-induced zinc spark is a novel biomarker of mouse embryo quality and early development
Source: Sci Rep. 2016 Mar 18;6:22772. doi: 10.1038/srep22772 (PMC4796984; doi:10.1038/srep22772)
Supplement: Supplementary Information [file srep22772-s1.pdf]

The fertilization-induced zinc spark is a novel biomarker of mammalian embryo quality and early development

Authors: Nan Zhang[1], Francesca E Duncan[1], Emily L. Que[2], Thomas V. O'Halloran[2, 3*] & Teresa K. Woodruff[1*]

Affiliations:

[1]Department of Obstetrics and Gynecology, Feinberg School of Medicine, Northwestern University, Chicago, IL 60611, USA.

[2]The Chemistry of Life Processes Institute, Northwestern University, Evanston, IL 60208, USA.

[3]Department of Chemistry and Department of Molecular Biosciences, Northwestern University, Evanston, IL 60208, USA

*To whom correspondence should be addressed: tkw@northwestern.edu, t-ohalloran@northwestern.edu

Supplementary Materials:

Fig. S1 shows a typical zinc spark induced by Ca-Iono and all the key parameters we used to analyze a zinc spark, which include amplitude (maximum peak height), integrated intensity (area under the curve), duration and rate of rise calculated as amplitude divided by the rising time.

We regularly tested the fertilization medium and culture conditions by performing standard IVF for 6 hours (materials and methods) and found that ~80% of the eggs could reach pronuclear-stage (PN) 8 hours after the initiation of fertilization (Fig. S2A, control group). To make sure that the eggs are fertilizable under the zinc imaging conditions, we also employed zona pellucida free (ZP free) eggs in our studies as removal of the zona pellucida is a common treatment that has been used to speed up sperm entry and counteract the deleterious effects of fluorescence imaging on the fertilization rate (*43, 44*). In our hands, we found that over 80% of the ZP free eggs could be fertilized during a 2-hour imaging period (Fig. S2A, -ZP group). We also confirmed that fertilization occurred based on the observation of multiple calcium transients similar to calcium oscillations reported by previous studies (Fig. S2B) (*33*). These findings suggest that the current imaging condition did not severely affect the health status of both eggs and sperms and the observation of single zinc spark during IVF is a physiological phenomenon.

Fig. S3 is a max intensity projection of zinc spark imaging following activation by 5μM Ca-Ionomycin. These resulting parthenotes were cultured for 120 hours and the developmental results were denoted on each egg. This data directly visualizes the close correlation between zinc spark profile and embryo development in mouse.

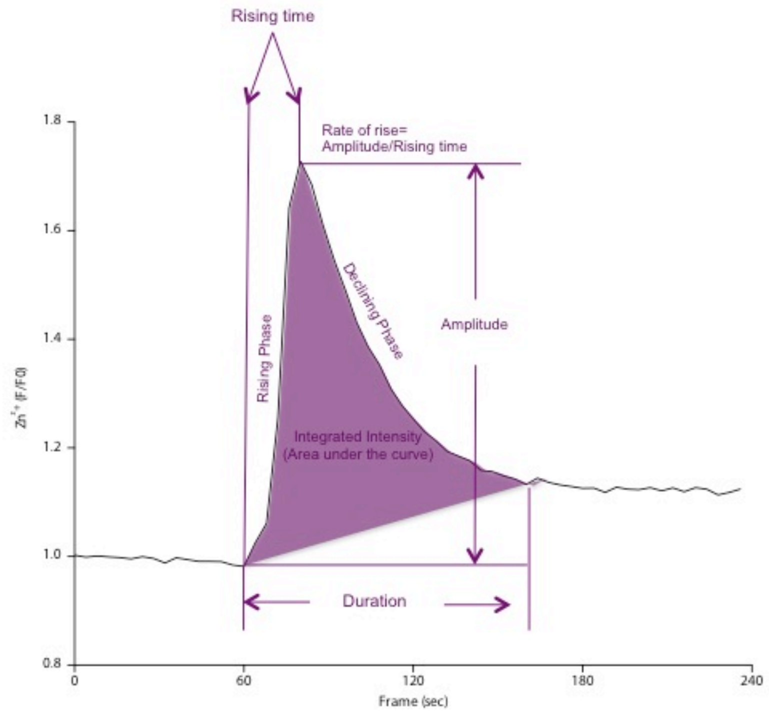

Fig. S1. Definition of key parameters of a representative zinc spark induced by 5μM Ca-Iono

Amplitude is a measurement from the base line to the highest point of the wave. Duration of a zinc spark, rising phase and declining phase are denoted in the figure. Integrated Intensity of the zinc spark is calculated as the area under the curve. Rate of rise is calculated as the amplitude divided by the rising time.

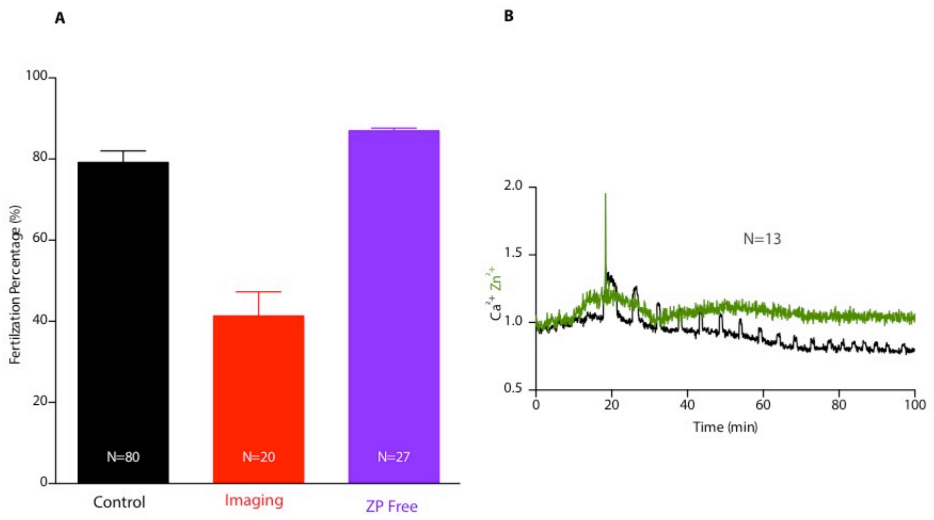

Fig. S2. Eggs are fertilizable under the optimized zinc imaging condition

(A) Fertilization rate of eggs under various conditions: Control group (Standard IVF without any treatments required by imaging), imaging group (Momentary serum free medium and 2-hour fluorescence imaging in the presence of FZ3) and ZP free group (IVF of ZP free eggs under the same condition as imaging group). (B) Representative time traces of $[Ca^{2+}]_i$ (green) and zinc sparks (pink) during IVF.

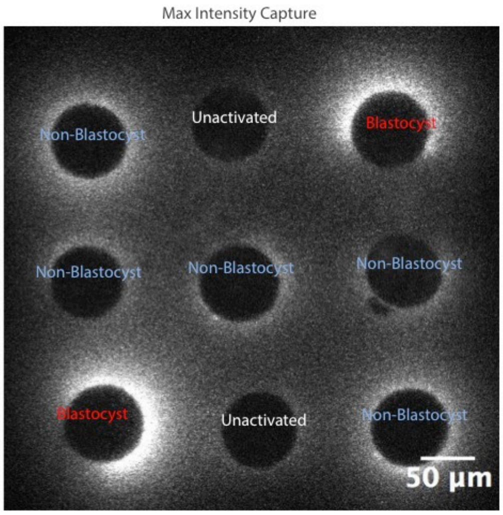

Fig. S3. Fluorescence intensity of zinc spark is closely related to the developmental outcome

Max intensity projection of zinc spark imaging following activation by 5μM Ca-Ionomycin (Movie S1). The developmental results were denoted on each egg.

Supplementary Figures

Fig. S1. Definition of key parameters of a zinc spark

Fig. S2. Fertilization rate under different imaging conditions

Fig. S3. Fluorescence intensity of the zinc spark is closely related to the developmental outcome

Supplementary Files

Movie S1. Ca-Iono induced zinc spark. (Scale bar 50 μm.)

Movie S2. Fertilization induced zinc spark. (The egg in the bottom right is presented in Fig. 2A. Scale bar 50 μm.)
